# Parasitic infections: A new frontier for PGD_2_ functions

**DOI:** 10.1016/j.crimmu.2024.100078

**Published:** 2024-05-25

**Authors:** Bruno L. Diaz, Christianne Bandeira-Melo

**Affiliations:** Laboratório de Inflamação, Instituto de Biofísica Carlos Chagas Filho, Universidade Federal do Rio de Janeiro, Av. Carlos Chagas Filho 373, Cidade Universitária, Rio de Janeiro, RJ, 21941-902, Brazil

## Abstract

Prostaglandin (PG)D_2_ is produced and/or triggered by different parasites to modulate the course of the infection. These findings position PGD2 as a therapeutic target and suggest potential beneficial effects of repositioned drugs that target its synthesis or receptor engagement. However, recent *in vivo* data may suggest a more nuanced role and warrants further investigation of the role of PGD2 during the full course and complexity of parasitic infections.

Prostaglandin (PG)D_2_ is one of the bioactive eicosanoids – 20 carbon lipid mediators produced by the metabolism of the polyunsaturated fatty acid Arachidonic Acid (AA) through different enzymatic steps. Once the cell is activated, the cytosolic Phospholipase A_2_ (cPLA_2_) hydrolyzes AA from phospholipids. The free AA is sequentially metabolized by a cyclooxygenase (COX) and a PGD synthase (PGDS) to produce PGD_2_. The particular isoform involved in each enzymatic step will depend on the type and activation status of the cell. In the immune system, PGD_2_ is generated in a biphasic fashion with an early phase dependent on the interplay of COX-1 and the hematopoietic (h)PGDS and dependent on a sharp increase in intracellular calcium that is characteristic of acute mast cell activation that parallels degranulation. A delayed PGD_2_ generation phase may occur in different cell types following COX-2 induction. The identity and/or intensity of the stimulus and the existence of cell priming may trigger the assembly of the multistep enzymatic machinery for the production of PGD_2_ in different subcellular compartments: sharp increases in intracellular calcium triggered by calcium ionophores or FcεRI crosslinking leads to perinuclear/ER production, while other physiological stimuli trigger the biogenesis of lipid bodies as the main loci for eicosanoid generation (Luna-Gomes et al., 2013; Rittchen and Heinemann, 2019) (Fig. 1a).

**Fig. 1:**
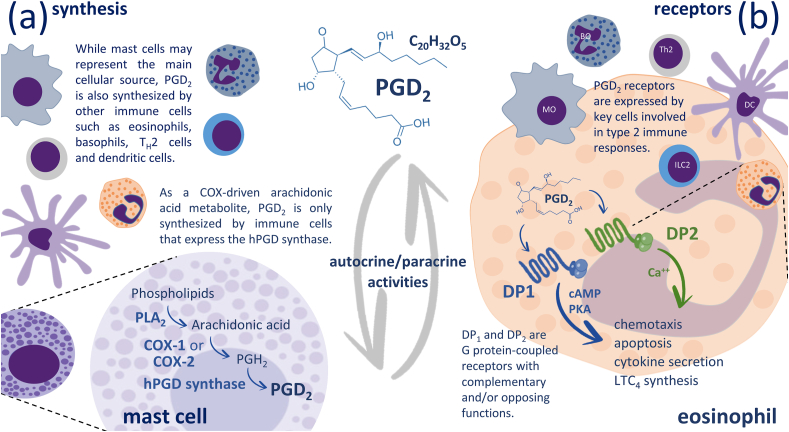
PGD_2_ synthesis and mechanism of action. (a) Arachidonic acid released from phospholipids by a Phospholipase (PL)A_2_ is metabolized by either the constitutive cyclooxygenase (COX)-1 or the inflammatory mediator inducible COX-2. Hematopoietic (h)PGD synthase is the final enzymatic step to produce PGD_2_ in immune cells such as mast cells, eosinophils, basophils, TH2 cells and dendritic cells. (b) Once produced PGD_2_ may act on two G-protein coupled receptors: DP1 and DP2, that trigger distinct signaling pathways that may act cooperatively or antagonistically to regulate different cell functions. Hence, PGD_2_ can modulate Allergic reactions and other type 2 immune responses.

Mast cells are usually recognized as the main source of PGD_2_ in the inflammatory milieu. However, several different cell types have been recognized that express hPGDS and produce biologically relevant PGD_2_ such as basophils, neutrophils, macrophages, and dendritic cells. Eosinophils have been initially identified as important targets of PGD_2_, also emerged as important sources of PGD_2_ with both autocrine and paracrine roles in allergy and parasitic infections (Lewis et al., 1982; Tanaka et al., 2000; Shimura et al., 2010; Luna-Gomes et al., 2011; Ugajin et al., 2011) (Fig. 1a).

PGD_2_ exerts its effects through the activation of two different receptors, DP1 and DP2, that couple to different signaling pathways. The outcome will depend on the relative expression levels of each receptor on the target cells and the intracellular context that may result in antagonistic or cooperative final effects. PGD_2_ can also be further metabolized to compounds such as 11β-PGF_2_α and J series prostanoids that retain the ability to bind and activate the DP2 receptor. Several PGD_2_ metabolites, particularly 15-deoxy-Δ^12,14^-PGJ_2_, are also known to activate PPAR receptors (Jandl and Heinemann, 2017; Fujino, 2021) (Fig. 1b).

The role of PGD_2_ in allergic reactions has been determined with a combination of pharmacological and genetic approaches and is reviewed elsewhere (Jandl and Heinemann, 2017; Rittchen and Heinemann, 2019). Several clinical trials targeting PGD_2_ receptors were started, but few resulted in clinically useful drugs, with the relevant exception of the beneficial effect in allergic rhinitis. This graphical review focus on the roles of PGD_2_, and its receptors, on infections by intracellular parasites and helminths.

Identification of eicosanoid production by nonmammalian species opened up the recognition of their potential role in parasitic infections (Bundy, 1985; Szkudlinski, 2000). Filarial worms produce eicosanoids that inhibit platelet aggregation and vasoconstriction ensuring their mobility in the vasculature, while eicosanoids increase the penetration of cercaria and are produced by adult *S. mansoni* worms (Fusco et al., 1985; Salafsky and Fusco, 1987). As illustrated in Fig. 2, Fig. 3, in addition to parasites possessing the enzymatic machinery to produce PGD_2_ for autocrine effects or on host cells, parasite infection can also trigger PGD_2_ generation by host cells. Irrespective of its cellular source, PGD_2_ seems to represent an important component of the interaction between host and parasites.

**Fig. 2:**
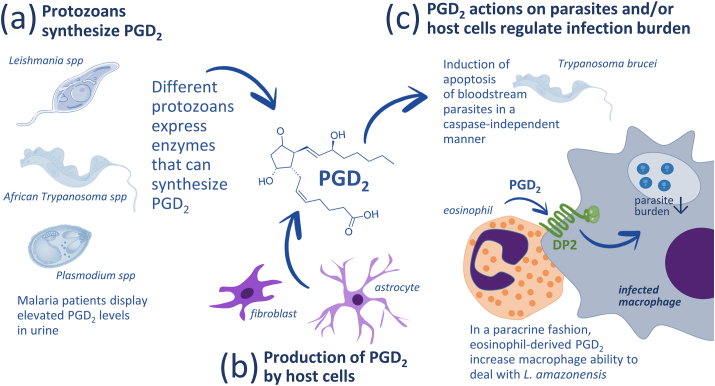
Participation of PGD_2_ in protozoan infection. (a) PGD_2_ is produced autonomously by different types of protozoan parasites and (b) its production can be triggered in immune cells in response to parasite-derived signals. (c) The subsequent increases in PGD_2_ levels in bodily fluids may impact the parasite and the host cells regulating parasite burden and symptoms.

**Fig. 3:**
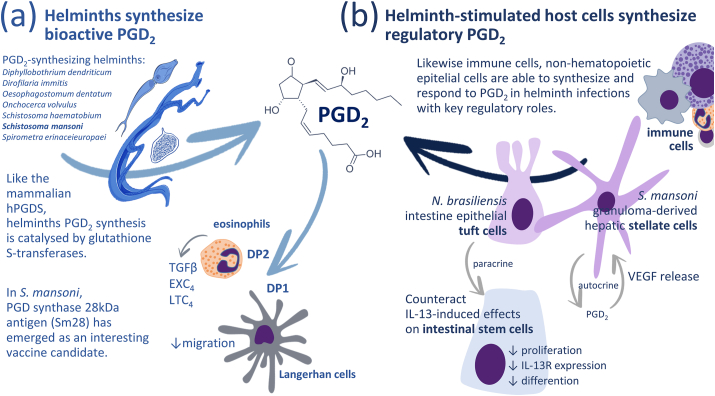
Role of PGD_2_ in helminth infection. (a) Helminths express a PGD synthase ortholog that can synthetize PGD_2_. (b) Helminths can trigger PGD_2_ production by host cell through activation of Pattern Recognition Receptors or direct tissue damage. The increase in PGD_2_ facilitates the parasite's escape from the host immune response and increases infection burden.

## 1 PGD_2_ in protozoan infections

PGD_2_ role in protozoan infections was initially inferred from the identification of elevated levels in cerebrospinal fluid of advanced sleeping sickness patients (Pentreath et al., 1990). PGD_2_ can be produced by *Trypanosoma brucei* (Kubata et al., 2000), the etiological agent of African Trypanosomiasis. *Leishmania* spp. also have the enzymatic machinery to produce prostanoids (Tavares et al., 2021) and PGD_2_ was identified as one of the products of *Leishmania donovani* (Kabututu et al., 2002) (Fig. 2a). PGD_2_ synthesis can also be triggered by products from this parasite in fibroblasts and astrocytes *in vitro* (Alafiatayo et al., 1994) (Fig. 2b). The ability to modulate eicosanoid metabolism *in vivo* correlates with disease progression and may be related to the associated immune suppression, fever, pain, and dysregulation of sleep/wake cycles (Fierer et al., 1984; Pentreath et al., 1990; Figarella et al., 2005). On the other hand, PGD_2_ may also have a role in directly regulating the parasite density through induction of cell death (Figarella et al., 2005). The complexity of PGD_2_ actions in trypanosomiasis can also be observed after treatment with its metabolite, 15-deoxy-Δ^12,14^-PGJ_2_, that can reduce inflammatory markers while increasing intracellular growth of *T. cruzi* in neonatal cardiomyocytes (Hovsepian et al., 2011) (Fig. 2c).

It is now clear that eicosanoid synthesis by either parasites themselves or parasite-stimulated host cells can participate in the protective response to eliminate or contain the parasites or be coopted to facilitate parasite survival and/or proliferation. For instance, PGD_2_ is also produced by *Plasmodium falciparum* and may contribute to pathogenesis and host-parasite interaction during malaria (Kilunga Kubata et al., 1998) (Fig. 2a). And, as illustrated in Fig. 2c, eosinophil-derived PGD_2_ can reduce *Leishmania amazonensis* burden in infected macrophages through paracrine activation of DP2 receptors (da Silva Marques et al., 2021). It is noteworthy that *Leishmania* spp express a PGF_2_α synthase ortholog that contributes to the pathogenicity through PGF_2_α generation from PGH_2_ (Alves-Ferreira et al., 2020), but may also have a role in the metabolism of PGD_2_ to reduce the anti-parasitic effects of host-derived PGD_2_. *T. cruzi* and *Leishmania* *spp*. can also take advantage of eicosanoid generation initiated by efferocytosis to promote the infection of the host (Freire-de-Lima et al., 2000; Ribeiro-Gomes et al., 2004).

## 2 PGD_2_ in helminthic infections

During parasitic infection, eicosanoids - derived from the parasite itself, immune cells and non-immune host cells - modulate different aspects of parasitic worms' interaction with their mammalian hosts, from penetration into the hosts organism to the modulation of the subsequent immune response and the expulsion of the parasites to reduce infection burden, but also to complete the parasite's life cycle and dissemination to other hosts (Fig. 3a).

PGD_2_ has been recognized as a product of parasitic worms such as *Schistosoma mansoni*, *Wuchereria bancrofti*, *Brugia malayi*, and *Trichinella spiralis* through similar catalytic steps and may act as a ligand of its cognate receptors on the mammalian host cells (Fig. 3a). *N. brasiliensis* infection induces PGD_2_ generation and activation of DP2 receptor that reduces the type 2 immune response and goblet cell hyperplasia resulting in less efficient worm expulsion (Oyesola et al., 2021). Meanwhile, PGD_2_ produced by a PGD synthase from *S. mansoni* can inhibit the migration of Langerhan cells from the host skin through activation of DP1 leading to increased parasite burden and egg-induced intestinal and liver damage (Angeli et al., 2001; Hervé et al., 2003). Lipids extracted from *S. mansoni* adults also trigger eosinophil activation and TGF-β production through DP1 activation (Magalhaes et al., 2019). These parasites can also co-opt the host cells to produce PGD_2_ through direct cell damage provoked by their movement through the host tissue or the release of factors that signal innate immune cells such as mast cells, eosinophils, and macrophages or nonhematopoietic cells to produce PGD_2_ and modulate the parasite infection (Kubata, 2007) (Fig. 3b). Hepatic stellate cells isolated from *S. mansoni*-infected livers convert to a myofibrobastic phenotype and respond to TGF-β with production of PGD_2_ that in turn activates the production of VEGF in an autocrine manner to promote tissue remodeling (Paiva et al., 2015). However, in an *in vivo* model of hepatic granulomatous reaction triggered by *S. mansoni* infection, PGD_2_ may play a protective role decreasing liver fibrosis via DP2 activation (Pezzella-Ferreira et al., 2023).

## 3 PGD_2_ and therapeutic perspectives in neglected parasitological diseases

**Fig. 4:**
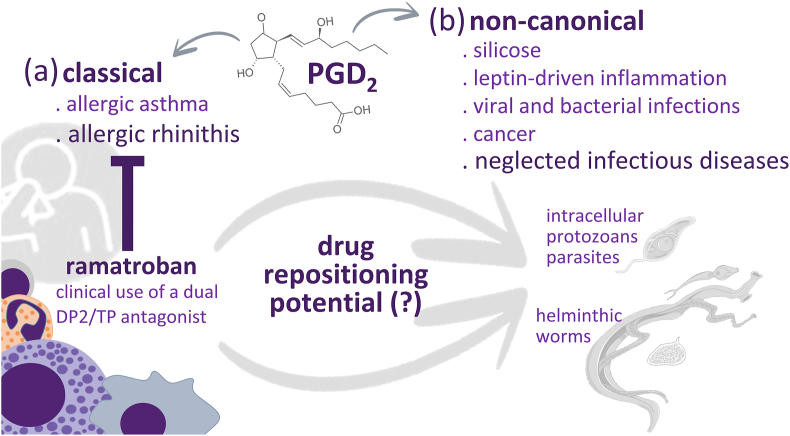
PGD_2_ suitability as a therapeutic target. (a) Antagonism of DP2 receptor has been shown to be a safe therapeutic approach to allergic conditions. (b) The use of DP2 antagonists has also been suggested for the treatment of diseases where PGD_2_ plays a non-canonical role, including neglected infectious diseases. Thus, repositioning of such drug may provide a relevant alternative for other conditions on which PGD_2_ is described to have a role. Due to the complex nature of parasite infections that include different evolutive forms of the parasites and an ever-changing immune response landscape, caution should be exercised so the therapeutic intervention is employed where and when it will produce the desired outcome.

Evidence for the role of PGD_2_ in parasitic infections has been demonstrated for many parasites. However, the exploration of PGD_2_ (and its receptors) as a potential target for the modulation of the pathogenesis associated with parasite infection in patients has been lacking. Antagonism of DP2 receptor antagonist has been in use for allergic conditions (Kupczyk and Kuna, 2017) and has also been proposed as a therapeutic approach for conditions where PGD_2_ plays a non-canonical role such as obesity, cancer, viral, and bacterial infections. Targeting PGD_2_ as potential therapy in parasitic diseases has also been proposed. Particularly relevant for schistosomiasis, Magalhães et al. showed that lipids isolated from adult worms of *S. mansoni* were able to activate TGF-β secretion by eosinophils through DP1 and TLR2 engagement. The capacity of PGD_2_ generated by *S. mansoni* adult worms to induce the synthesis of a major profibrotic mediator supplied the rationale to target PGD_2_ as a potential therapy in parasitic diseases (Magalhaes et al., 2019). Later, Coakley et al. proposed a potential exosome-dependent mechanism for the delivery of PGD_2_ to eosinophils in a similar fashion that was demonstrated for the delivery of TLR2 ligands. DP1 activation by PGD_2_ carried by exosomes would in turn stimulate TGF-β synthesis and a pro-fibrotic role by eosinophils in schistosomiasis (Coakley et al., 2019). This potential profibrotic role of PGD_2_ was also highlighted by Acharya et al. with the description of a pro-fibrotic loop involving PGD_2_-eosinophils-TGF-β-hepatic stellate cells-PGD_2_ (Acharya et al., 2022). Although hepatic fibrosis is a major target in schistosomiasis, the role of PGD_2_ in this condition in the *in vivo* setting goes beyond DP1 engagement (Hervé et al., 2003). Through DP2 activation, PGD_2_ may provide a dominant anti-fibrotic effect in hepatic granulomas in schistosomiasis (Pezzella-Ferreira et al., 2023). DP2 activation also plays a potential host-protective role through the reduction of leishmania parasite proliferation inside macrophages (da Silva Marques et al., 2021). Thus, targeting PGD_2_, especially through the repositioning of the DP2 receptor antagonist, Ramatroban, may aggravate the disease. Thus, care should be exercised in targeting PGD_2_ in parasitic diseases as it may also play a protective role for the host (Fig. 4).

## Declaration of competing interest

The authors declare the following financial interests/personal relationships which may be considered as potential competing interests: Bruno L. Diaz reports financial support was provided by Carlos Chagas Filho Foundation for Research Support of Rio de Janeiro State. Christianne Bandeira-Melo reports financial support was provided by Carlos Chagas Filho Foundation for Research Support of Rio de Janeiro State. Christianne Bandeira-Melo reports financial support was provided by 10.13039/501100003593National Council for Scientific and Technological Development. Bruno L. Diaz reports financial support was provided by 10.13039/501100003593National Council for Scientific and Technological Development. If there are other authors, they declare that they have no known competing financial interests or personal relationships that could have appeared to influence the work reported in this paper.

## Data Availability

No data was used for the research described in the article.
